# Supplementary material for: Causal Relationship between Aging and Anorexia Nervosa: A White-Matter-Microstructure-Mediated Mendelian Randomization Analysis
Source: Biomedicines. 2024 Aug 16;12(8):1874. doi: 10.3390/biomedicines12081874 (PMC11351342; doi:10.3390/biomedicines12081874)
Supplement: Supplementary file 1 [file biomedicines-12-01874-s001.zip › Supplementary Figure/Figure S8/Leaveoneout plot of MR analysis for TL to ubm-b-1717.pdf]

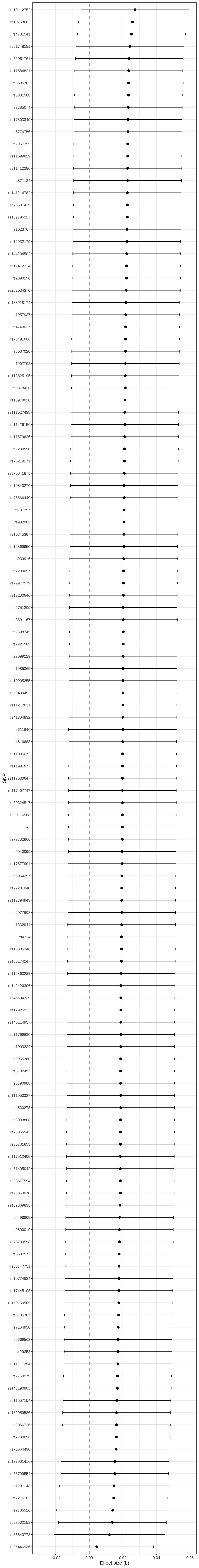

rs10112752
rs10769683
rs4731541
rs61748181
rs56061761
rs11584821
rs6536702
rs6881568
rs3785074
rs17803849
rs6776756
rs2967355
rs11699829
rs11412296
rs871134
rs141214282
rs73581419
rs139795227
rs1023767
rs12932179
rs144204502
rs12412214
rs9398196
rs202034370
rs188918174
rs1957937
rs4743037
rs78491606
rs6007020
rs1907702
rs113525195
rs9878436
rs16978028
rs111527438
rs11426156
rs11579626
rs2230590
rs76219171
rs376641875
rs10840270
rs76666449
rs131797
rs932002
rs10845387
rs12369950
rs939916
rs7209057
rs79977579
rs13230646
rs6751209
rs3891167
rs2538745
rs7221585
rs7099229
rs1985369
rs10905255
rs59409453
rs11212631
rs41304832
rs611646
rs4616688
rs11085072
rs11991877
rs117630647
rs117407747
rs80324517
rs80116508
All
rs77732866
rs9940099
rs17677991
rs6054257
rs77231040
rs112394943
rs2977608
rs1332941
rs4724
rs10805346
rs185174247
rs116863223
rs142426306
rs45604339
rs12925933
rs145114957
rs11769630
rs1003322
rs9955360
rs8102497
rs6790988
rs11950327
rs4530278
rs3093888
rs76065543
rs66731853
rs117512405
rs61405042
rs28577594
rs28363070
rs139669835
rs4498805
rs9600019
rs73730598
rs6587577
rs55747751
rs10774624
rs17445108
rs150150565
rs8105767
rs7164950
rs6669563
rs429358
rs11117354
rs2763979
rs143190905
rs11557154
rs182059586
rs2056726
rs7790856
rs75664430
rs137901416
rs56799554
rs1291143
rs2276182
rs7705526
rs28502153
rs35640778
rs35446936
SNP
-0.02
0.00
0.02
0.04
0.06
Effect size (b)
